# The development and flux of the University of Minnesota Survivorship Program: progress, challenges, and opportunities

**DOI:** 10.1007/s11764-023-01520-z

**Published:** 2024-01-31

**Authors:** Tara J. Rick, Jill Lee, Sasha Skendzel, Kaia Verich, Ashley Schempp, Char Napurski, Holly Kreuser, Lucie Turcotte, Shernan Holtan, Karim Sadak, Anne Blaes

**Affiliations:** 1 Department of Medicine, Division of Hematology, Oncology, and Transplantation, University of Minnesota, Minneapolis, MN USA; 2 M Health Fairview, Minneapolis, MN USA; 3 Masonic Cancer Center, University of Minnesota, Minneapolis, MN USA; 4 Department of Pediatrics, Division of Hematology/Oncology, University of Minnesota, Minneapolis, MN USA

**Keywords:** Cancer Survivorship Program, Long-term follow-up, Program evaluation, Cancer, Cancer survivorship

## Abstract

**Abstract:**

For the past 30 years, the University of Minnesota’s Cancer Survivorship Program has been dedicated to providing exceptional care to patients who have lived the cancer experience. Our model is consultative, risk-stratified, and oncologist-led but executed predominately by advanced practice providers. Care is personalized and serves three survivor populations: children, adults, and patients who received BMT with over 500 new patients evaluated annually. As guidelines and survivorship standards have changed, our clinical programs have evolved from a focus on survivorship care plans to supportive care. The program offers a wide range of supportive services from acupuncture to nutritional services as well as several educational programs for patients. The program has a strong research legacy, notably as the birthplace of research that led to the Children’s Oncology Group Guidelines as well as advancements in cardio-oncology and frailty after bone marrow transplantation. In 2021, we hosted the first annual Survivorship Research Forum, providing the opportunity and space for experts across disciplines to exchange ideas on a broad range of survivorship topics not possible at other national cancer-related conferences. With successes and challenges, we have identified opportunities for growth as our program continues to evolve and grow in our goal to improve cancer outcomes along a wide spectrum of physical, emotional, functional, and social dimensions.

**Implications for Cancer Survivors:**

The University of Minnesota Cancer Survivorship Program provides care, education, and research opportunities for patients across the cancer continuum.

## Introduction

Approximately two decades ago, the Institute of Medicine (IOM) released the report “From Cancer Patient to Cancer Survivor” highlighting the need to prevent cancer recurrence, screen for new cancers, provide surveillance for long-term physical and emotional late effects, and facilitate coordination of care between specialists and primary care providers [1]. Since then, the University of Minnesota (UMN), a public research university, has had multiple iterations of cancer survivorship care in alignment with changes in our understanding of cancer survivorship care and associated guidelines. UMN health care system includes a referral center in the Twin Cities with several hospitals and clinics in the surrounding metropolitan and rural areas. The UMN Cancer Survivorship Program sees over 500 new long-term follow-up (LTFU) patients a year, predominately brain tumors, leukemias, lymphomas, gynecologic, and breast cancer survivors, although all tumor types are served in our program. Although most of our cancer survivorship population identifies as non-Hispanic White, our community has a diverse population encompassing various racial and ethnic backgrounds including Somali, Mexican, Hmong, Karin, and Ethiopian immigrant populations. The University of Minnesota serves to provide high-quality education, research, and medical care to the people of Minnesota and the upper Midwest.

##  Historical perspective

The Cancer Survivorship Program at UMN has a rich history that spans over three decades. It was initially founded to serve pediatric cancer survivors and was the birthplace of the Long-term Follow-Up Study (now the Childhood Cancer Survivor Study—CCSS) in 1994 led by Dr. Leslie Robison. While now located at St. Jude, CCSS (NCI U24 CA55727) has been instrumental in advancing the understanding of the long-term health outcomes of childhood cancer survivors and led to the development of the Children’s Oncology Group (COG) guidelines for long-term follow-up care. After the initial reports of CCSS in 2003, the long-term follow-up (LTFU) clinic, via a consultative model for pediatric cancer survivors, was established at UMN [2]. The program expanded to adult cancer survivors in 2009, after the release of the IOM report [1], and subsequently to bone marrow transplantation (BMT) after participation in studies addressing the utilization of survivorship care plans in BMT [3].

## Clinical services

### Care delivery model and populations served

The cancer survivorship program provides comprehensive care that addresses the physical, emotional, and psychological needs of cancer survivors and their families of all ages. There are three distinct clinical services: childhood, adult, and adult BMT services. The clinical services all share the same goal; however, the services function slightly differently to cater to the unique needs of each patient population. The dedicated cancer survivorship clinics operate on a consultative, oncologist-led, risk-stratified model for patients who are greater than 5 years out from completing cancer treatment or 1 and 2 years out from bone marrow transplantation. Survivorship care plans (SCPs) are created for the initial visit and distributed electronically to the patient, referring provider, and primary care provider through our institution’s Electronic Health Record (EHR) vendor, Epic. SCPs are integrated into the EHR as a distinct document under the diagnosis in the problem list or integrated into the visit note. Some treatment data is automatically populated via EHR tools, while nursing staff finalize the treatment summary data and the clinicians review and complete the care plan by individualizing it to the specific needs of the patient.

In the childhood cancer service, which includes children who have had BMT, patients are automatically funneled into LTFU by their treating physician. The program entails an annual visit. Afterwards, they are invited to enroll in a research database available for ongoing research and invitations to future prospective studies. Childhood cancer survivors are followed longitudinally into adulthood by a team of providers—pediatric hematologist/oncologist, advanced practice provider, med/peds physician, research nurse, and social work, being seen on the pediatric campus until ages 25–29 years, when they transfer care to the adult campus. Between 2018 and 2022, there were 1868 total LTFU visits with an average of 61 new patients/year, and the average age at initial visit was 17 years. The SCP completion rate during this period across the childhood service was 99%. There are an average of two supportive care referrals per initial visit.

In the adult cancer survivorship service, patients are referred by their treating oncologist, primary care provider, or may self-refer 5 years after cancer diagnosis. A cancer survivorship (also called LTFU) visit is either a one-time consultative visit or longitudinal, depending on the needs of the patient and risk stratification. In 2016, with American College of Surgeon’ Commission on Cancer (CoC) accreditation standards and guideline modifications, cancer survivorship care was modified to include cancer survivorship care earlier in the cancer continuum, as an “end-of-treatment” visit provided by advanced practice providers (APPs) within disease-specific groups. Historically, the program has been focused on patients who received curative intent therapy, however, as the applied definition of cancer survivorship broadens, so does the scope of our program, and increasingly, we have more patients living with cancer being referred to adult cancer survivorship. Between 2018 and 2022, there were 1200 total patient visits in the survivorship clinic with approximately 123 new patients/year; average age at initial visit was 61 years. Additionally, the adult program provides 1100+ SCPs annually to patients at the end of their treatment, achieving 60% compliance per previous CoC definition. There is an average of 1 supportive care referral per initial visit.

For adult BMT patients, dedicated survivorship visits are scheduled at specific intervals, at year one and two after BMT. The care is provided by a dedicated APP. There has been a Transplant Late-effects Clinic (TLC) for patients who are referred from an outside institution. In the adult BMT services, there were 650 total patient visits between 2018 and 2022, with an average of 105 new patients/year. The average age at initial visit was 59 years. SCP completion rate was 56%. There is an average of 0.5 supportive care referrals per initial visit. Survivorship services were reduced to paused across the services during the COVID-19 pandemic and have now restarted.

### Accreditation and standards

The Cancer Survivorship Program received initial accreditation from the CoC in 1987, accreditation at the University site in 2018 and full network accreditation in 2022. In 2016, the CoC standard 3.3 mandated that all individuals undergoing curative therapy should be provided with a comprehensive care summary and follow-up plan. With this mandate, we were able to leverage institutional support for survivorship and supportive care services, additional nursing support, as well as a culture shift in addressing the supportive care needs of patients both during and after therapy. The CoC implemented updated standards 4.8 in 2020, and the standards shifted from survivorship care plans to the process of delivering high-quality survivorship care. We have focused on building a multidisciplinary team to implement a comprehensive survivorship program and team that cater to the unique needs of cancer patients (Table 1). The committee meets quarterly, reviews metrics, and outlines goals for future and ongoing supportive care needs. For example, each year, the program selects three services to review. Examples of such program review include frequency of acupuncture, cancer rehabilitation, fertility and Physical Medicine and Rehabilitation referrals as well as treatment summary activity. These comprehensive services aim to address the diverse needs of cancer survivors and promote overall wellbeing and quality of life.

**Table 1:** Cancer survivorship and supportive services

Cancer Survivorship Program (childhood, adult, and adult BMT services)*	A dedicated oncology visit which includes a cancer treatment summary and personalized care plan, screening for late effects of cancer treatment, referral to supportive care services where applicable, and provide tailored education and resources. A brief psychosocial assessment with a social worker (childhood program)
Acupuncture	Acupuncture provided by medical doctors, chiropractic doctors and licensed acupuncturists located in our medical faculty to treat ailments such as pain, anxiety, neuropathy, and promote overall wellbeing
Cardio-oncology	Cardiologists trained to mitigate and treat cardiac complications of cancer treatment
Cancer rehabilitation*	Program includes a physical medicine and rehabilitation (PM&R) physician, physical and occupational therapist, and speech language pathologists with training and experience working with cancer survivors
Comprehensive weight management	A comprehensive program that pairs evidence-based medicine and individual goals and lifestyle to create a personalized care plan. A dedicated care team includes a provider, dietician, and a health coach, with referrals available to other care specialists as needed
Fertility services	Education and resources for referral to urology or reproductive endocrinology
Financial/employment/insurance*	Social workers and financial counselors assist with questions regarding insurance, employment, and other financial concerns
Genetic counseling/Cancer Risk Management Program	Genetic counselors help families make informed decisions about genetic testing and help interpret test results. Cancer Risk Management Program is designed to help patients evaluate their risk of developing cancer and work with them to manage their cancer risk
Health and wellbeing	Available resources include the Earl E. Bakken Center for Spirituality & Healing and the Taking Charge of your Survivorship website which offer safe and reliable information of topics such as mindfulness, nutrition, mental health, and others to care for the body, mind, and spirit
Lymphedema therapy*	Certified lymphedema therapists provide a customized treatment plan which may include exercises, education, and manual techniques
Nicotine cessation	Referral to Minnesota Quit Partner which offers free nicotine cessation counseling and other services as well as pharmacy consults for medication management
Oncology nutrition*	Oncology dieticians specialize in optimizing nutritional intake and achieving treatment goals
Oncology psychology*	Referral to an oncology psychologist to address the psychological, behavioral, emotional, and social issues that arise with a cancer diagnosis including neuropsychiatric testing for chemotherapy-related cognitive impairment
Palliative care	Care provided by a team of doctors, nurses, social workers, and other specialists to provide support for those living with cancer
Sexual health	Resources include Thrive videos and informational brochures, visits with our survivorship providers, and referral to the Institute for Sexual and Gender Health
Social work*	Licensed social worker referrals for comprehensive patient-centered care by supporting emotional health and assist with any barriers to receiving care
Other specialty referral services	audiology, dermatology, endocrinology, gastroenterology, pulmonary, rheumatology, sleep medicine, obstetrics/gynecology, urology

*Member of CoC Cancer Survivorship Committee

While the focus has shifted away from SCPs, we persist in utilizing them as a fundamental element of our program. Previously, when SCPs took center stage, they diverted attention away from other crucial programming and objectives. However, we continue to find value in SCPs as they facilitate discussions about survivorship care and effectively convey follow-up care information to patients and their caregivers, especially with a consultative model. Utility is assessed periodically and the patient feedback about their usefulness has been varied. Some patients exhibit great motivation, while others feel that SCPs are introduced too late (when introduced 5 years from active treatment). An area of opportunity to enhance SCP utility may be introducing cancer survivorship earlier in the cancer continuum.

### Survivorship care delivery model

The care within the program is provided by a team which includes at least five physicians, three APPs, and three nurses who work collaboratively to deliver comprehensive and personalized care to cancer survivors. Each distinct clinical service (childhood, adult, and adult BMT survivorship) has a dedicated medical director, who ensures the quality and effectiveness of the program and a dedicated oncology APP champion with leadership on the team and who is passionate about supporting the needs of cancer survivors. Nursing staff play a crucial role in the success of our cancer survivorship program by supporting patients and other team members by completing cancer treatment summaries, care coordination, and triaging patient concerns. Pediatric visits also include a psychosocial assessment with a social worker. Lastly, we have a survivorship program manager who oversees every aspect of our operations, including clinical and educational endeavors. Hence, the allocation of staff is determined by the unique needs of each service. However, at minimum, every service ensures an oncologist and an oncology APP champion with support from nursing.

As discussed above and summarized in Table 1, we work closely with a multidisciplinary team to optimize the care of our patients. Key specialists include dedicated cardio-oncologists, an oncology physical medicine and rehabilitation physician, an oncology psychologist, an oncology nutritionist, and a clinical nurse specialist in Cancer Risk Management. Also available are supportive services such as acupuncture, dedicated oncology physical and occupational therapy, comprehensive weight management, fertility services, financial/employment/insurance assistance, genetic counselors, and nicotine cessation. We offer additional wellness resources available to patients through our Center for Spirituality and Healing. Lastly, for sexual health, we have a psychologist from the UMN Institute for Sexual and Gender Health with a focus on cancer survivors as well as an APP within survivorship with a special interest in sexual health in cancer survivors.

### Timing of survivorship care

The timing of care delivery of our pediatric model was built based on the LTFU/CCSS cohorts, with entry criteria of 5 or more years following treatment. Similarly, our adult program was designed by the same approach, recognizing that patients are closely monitored by the primary oncology team during the initial 5 years. However, as previously mentioned, we have begun utilizing survivorship care at the end of treatment for adult patients (surgery/chemotherapy/radiation) to introduce survivorship care, SCPs, resources, and educational opportunities earlier in the care continuum. Adult BMT patients receive SCPs at the end of treatment, specifically at hospital discharge after transplant, and are followed by dedicated survivorship visits at their protocol driven anniversary visits at year one and two.

### Funding

The clinicians and supportive care service providers bill for their visits. Philanthropy funds additional services such as social work and education. The Masonic Cancer Center, along with the Divisions (Division of Pediatric Hematology and Oncology and Division of Hematology, Oncology, and Transplantation) provided initial startup research infrastructure funding including database support and research coordinator. Currently, research infrastructure is primarily supported by research grants and philanthropic contributions. This support has enabled the clinic to provide comprehensive care to patients and conduct research to improve outcomes for those affected by cancer. The clinic’s commitment to long-term follow-up care ensures that patients receive ongoing support and monitoring, even after their initial treatment has ended.

### Key performance indicators

Compliance with CoC and National Accreditation Program for Breast Centers (NAPBC) survivorship standards is currently a primary measure of success in the adult program. As mentioned above, the multidisciplinary survivorship committee meets quarterly to evaluate the program. The key performance indicators evaluated are patient volumes, volume of participants in survivorship educational programming, growth of supportive care services (services offered and volume), expansion of services to patients with malignant hematology diagnosis, brain and spinal cord tumors, and individuals living with advanced disease.

## Educational program

Our Cancer Survivorship Program offers patient- and provider-facing educational opportunities. For the past 16 years, we have been hosting an in person annual Cancer Survivorship Conference with over 400 patients, caregivers, and health care providers participating each year. We also conduct a virtual Thrive Education Webinar Series annually, providing additional opportunities for survivors to enhance their well-being. Recordings of educational sessions are available on our website, and in 2022, there were nearly 2000 viewings of recorded material [4]. Our educational opportunities are available free of charge and are funded through grassroots efforts, including grants and donations to the program such as The National Childhood Cancer Society which supported the launch of the Cancer Survivorship Conference and provides annual support and facilitates collaboration with community-based programs. An additional partner is the Masonic Cancer Center Office of Community Outreach and Engagement (COE) which focuses on community-based cancer survivorship programing to diverse communities across Minnesota to reduce health disparities. COE has ongoing relationships with community organizations and non-profits to target some of the state’s largest, unique populations including Somali, Hmong, and American Indian tribes. In 2022, the COE team participated in 64 events reaching 31,803 participants including 694 cancer screenings. 89% of these events were composed of diverse audiences, an estimated 28,000+ attendees identified as other than non-Hispanic White.

Our institution has recently started exploring opportunities to support caregivers, as we recognize they are the “secondary” survivors. Collaboration with the American Cancer Society and a local nonprofit has led to caregiver focus groups, impactful videos shining a focus on their invaluable role, and inclusion of caregiver topics in survivorship related education events to delve deeper into this aspect of survivorship. Alongside the patient-oriented opportunities, we also offer monthly continuing medical education opportunities internally for physicians, APPs, nurses, and other allied staff through our UMN Cancer Survivorship Forum. Cancer survivorship visits are integrated into the clinical training of MDs and APPs in training.

## Research activities

As an academic program, we have ongoing research activities housed in the Masonic Cancer Center’s Screening, Prevention, Etiology, and Cancer Survivorship (SPECS) Program, an NCI designated cancer center. SPECS offers a dynamic platform for research working groups (pediatrics, adult, and pediatric epidemiology) and aims to support cutting-edge research through mentorship and grant funding, as well as an integration of MDs, APPs, PhD scientists with epidemiology, health disparity, and health policy research backgrounds. Research databases exist for all childhood cancer survivors diagnosed at our center and BMT survivors, with multiple cohorts of adult survivors (gyn onc, breast cancer). An integrated research team consisting of a project manager, RNs, and clinical research associates facilitates ongoing supportive care research activities with additional collaboration from the MCC Clinical Trials Office for interventional trials supporting cancer survivors. These efforts have led to $10 million in grant funding in 2022 and multiple high-impact publications examining second cancers, cardio-oncology, and transitions of care, for example (Table 2). Research efforts have advanced understanding of second cancer in childhood and young adult survivors, informing Children’s Oncology Group guidelines [5]. Research has also informed cardio-oncology guidelines and has led to new initiatives on HPV vaccinations in high-risk groups and examined the use of frailty assessments in BMT patients [6, 7]. Innovation in survivorship research includes development of a social care connection platform that utilizes innovative electronic health record technology to screen newly diagnosed patients, assess their social determinants of health, and connect them with tailored resources early on to provide supportive care.

**Table 2:** Sample of publications related to cancer survivorship research within the University of Minnesota 2018–2023.*Highlight Masonic Cancer Center member

**2018**	
Nyitray AG, Hicks JT, Hwang LY, Baraniuk S, White M, Millas S, Onwuka N, Zhang X, Brown EL, **Ross MW** , Chiao EY. A phase II clinical study to assess the feasibility of self and partner anal examinations to detect anal canal abnormalities including anal cancer. Sex Transm Infect. 2018 MAR 01; 94(2):124-130. DOI: 10.1136/sextrans-2017-053283. 2017 Aug 23. PubMed PMID: 28835533.	
Naber SK, **Kuntz KM** , Henrikson NB, Williams MS, Calonge N, Goddard KAB, Zallen DT, Ganiats TG, Webber EM, Janssens ACJW, van Ballegooijen M, Zauber AG, Lansdorp-Vogelaar I. Cost Effectiveness of Age-Specific Screening Intervals for People With Family Histories of Colorectal Cancer. Gastroenterology. 2018 JAN 01; 154(1):105-116.e20. DOI: 10.1053/j.gastro.2017.09.021. 2017 Sep 28. PubMed PMID: 28964749.	
**Joseph AM** , Rothman AJ, Almirall D, Begnaud A, Chiles C, Cinciripini PM, **Fu SS** , Graham AL, Lindgren BR, Melzer AC, Ostroff JS, Seaman EL, Taylor KL, Toll BA, Zeliadt SB, Vock DM. Lung Cancer Screening and Smoking Cessation Clinical Trials. SCALE (Smoking Cessation within the Context of Lung Cancer Screening) Collaboration. Am. J. Respir. Crit. Care Med.. 2018 JAN 15; 197(2):172-182. DOI: 10.1164/rccm.201705-0909CI. PubMed PMID: 28977754; PMCID: PMC5768904.	
**Poynter JN** , Richardson M, Roesler M, Krailo M, Amatruda JF, Frazier AL. Family history of cancer in children and adolescents with germ cell tumours: a report from the Children’s Oncology Group. Br. J. Cancer. 2018 JAN 01; 118(1):121-126. DOI: 10.1038/bjc.2017.358. 2017 Oct 24. PubMed PMID: 29065103; PMCID: PMC5765220.	
Scott MC, Temiz NA, Sarver AE, LaRue RS, Rathe SK, Varshney J, Wolf NK, **Moriarity BS** , O'Brien TD, **Spector LG** , **Largaespada DA** , **Modiano JF** , **Subramanian S** , **Sarver AL** . Comparative Transcriptome Analysis Quantifies Immune Cell Transcript Levels, Metastatic Progression, and Survival in Osteosarcoma. Cancer Res.. 2018 JAN 15; 78(2):326-337. DOI: 10.1158/0008-5472.CAN-17-0576. 2017 Oct 24. PubMed PMID: 29066513; PMCID: PMC5935134.	
Van Dyke AL, Langhamer MS, Zhu B, Pfeiffer RM, Albanes D, Andreotti G, Beane Freeman LE, Chan AT, Freedman ND, Gapstur SM, Giles GG, Grodstein F, Liao LM, Luo J, Milne RL, Monroe KR, Neuhouser ML, **Poynter JN** , Purdue MP, Robien K, Schairer C, Sinha R, Weinstein S, Zhang X, Petrick JL, et al. Family History of Cancer and Risk of Biliary Tract Cancers: Results from the Biliary Tract Cancers Pooling Project. Cancer Epidemiol. Biomarkers Prev.. 2018 MAR 01; 27(3):348-351. DOI: 10.1158/1055-9965.EPI-17-1003. 2018 Jan 16. PubMed PMID: 29339358; PMCID: PMC5835211.	
Petzel SV, **Isaksson Vogel R** , Cragg J, McClellan M, Chan D, Jacko JA, Sainfort F, **Geller MA** . Effects of web-based instruction and patient preferences on patient-reported outcomes and learning for women with advanced ovarian cancer: A randomized controlled trial. J Psychosoc Oncol. 2018 MAY 23; :1-17. DOI: 10.1080/07347332.2018.1457125. 2018 May 23. PubMed PMID: 29791275.	
**Khariwala SS (CC)**, **Hatsukami DK** , **Stepanov I (CC)**, Rubin N, **Nelson HH** . Patterns of Tobacco Cessation Attempts and Symptoms Experienced Among Smokers With Head and Neck Squamous Cell Carcinoma. JAMA Otolaryngol Head Neck Surg. 2018 JUN 01; 144(6):477-482. DOI: 10.1001/jamaoto.2018.0249. PubMed PMID: 29800964; PMCID: PMC6145745.	
**Turcotte LM** , **Neglia JP** , Reulen RC, Ronckers CM, van Leeuwen FE, Morton LM, Hodgson DC, Yasui Y, Oeffinger KC, Henderson TO. Risk, Risk Factors, and Surveillance of Subsequent Malignant Neoplasms in Survivors of Childhood Cancer: A Review. J. Clin. Oncol.. 2018 JUL 20; 36(21):2145-2152. DOI: 10.1200/JCO.2017.76.7764. 2018 Jun 6. PubMed PMID: 29874133; PMCID: PMC6075849.	
Kehm RD, **Spector LG** , **Poynter JN** , Vock DM, Altekruse SF, Osypuk TL. Does socioeconomic status account for racial and ethnic disparities in childhood cancer survival? Cancer. 2018 OCT 15; 124(20):4090-4097. DOI: 10.1002/cncr.31560. 2018 Aug 20. PubMed PMID: 30125340; PMCID: PMC6234050.	
Yzer M, Rhodes K, McCann M, Harjo J, **Nagler RH** , LoRusso SM, **Gollust SE** . Effects of cultural cues on perceptions of HPV vaccination messages among parents and guardians of American Indian youth. Prev Med. 2018 OCT 01; 115:104-109. DOI: 10.1016/j.ypmed.2018.08.021. 2018 Aug 25. PubMed PMID: 30153440.	
**Williams LA** , Pankratz N, Lane J, Krailo M, Roesler M, Richardson M, Frazier AL, Amatruda JF, **Poynter JN** . Klinefelter syndrome in males with germ cell tumors: A report from the Children's Oncology Group. Cancer. 2018 OCT 01; 124(19):3900-3908. DOI: 10.1002/cncr.31667. 2018 Oct 6. PubMed PMID: 30291793; PMCID: PMC6241518.	
Malagón T, **Kulasingam S** , Mayrand MH, Ogilvie G, Smith L, Bouchard C, Gotlieb W, Franco EL. Age at last screening and remaining lifetime risk of cervical cancer in older, unvaccinated, HPV-negative women: a modelling study. Lancet Oncol.. 2018 DEC 01; 19(12):1569-1578. DOI: 10.1016/S1470-2045(18)30536-9. 2018 Nov 1. PubMed PMID: 30392810.	
Rogers CR, Rovito MJ, Hussein M, Obidike OJ, **Pratt R** , Alexander M, Berge JM, Dall'Era M, Nix JW, Warlick C. Attitudes Toward Genomic Testing and Prostate Cancer Research Among Black Men. Am J Prev Med. 2018 NOV 01; 55(5S1):S103-S111. DOI: 10.1016/j.amepre.2018.05.028. PubMed PMID: 30670195; PMCID: PMC6352989.	
**2019**	
**Parsons HM** , Muffly L, Alvarez EM, Keegan THM. Does Treatment Setting Matter? Evaluating Resource Utilization for Adolescents Treated in Pediatric vs Adult Cancer Institutions. J. Natl. Cancer Inst.. 2019 MAR 01; 111(3):224-225. DOI: 10.1093/jnci/djy123. PubMed PMID: 30053066.	
**Parsons HM** , Penn DC, Li Q, Cress RD, Pollock BH, Malogolowkin MH, Wun T, Keegan THM. Increased clinical trial enrollment among adolescent and young adult cancer patients between 2006 and 2012-2013 in the United States. Pediatr Blood Cancer. 2019 JAN 01; 66(1):e27426. DOI: 10.1002/pbc.27426. 2018 Sep 6. PubMed PMID: 30256525; PMCID: PMC6249090.	
Tanner LR, **Hooke MC** . Improving body function and minimizing activity limitations in pediatric leukemia survivors: The lasting impact of the Stoplight Program. Pediatr Blood Cancer. 2019 MAY 01; 66(5):e27596. DOI: 10.1002/pbc.27596. 2019 Jan 4. PubMed PMID: 30609245.	
**Vogel RI** , Niendorf K, Petzel S, Lee H, **Teoh D** , **Blaes AH** , **Argenta P (CM)**, Rivard C, **Winterhoff B** , Lee HY, **Geller MA** . A patient-centered mobile health application to motivate use of genetic counseling among women with ovarian cancer: A pilot randomized controlled trial. Gynecol. Oncol.. 2019 APR 01; 153(1):100-107. DOI: 10.1016/j.ygyno.2019.01.019. 2019 Feb 1. PubMed PMID: 30718125.	
**Teoh D** , Shaikh R, Schnaith A, **Lou E (CM)**, **McRee AL** , **Nagler RH** , **Vogel RI** . Evaluation of graphic messages to promote human papillomavirus vaccination among young adults: A statewide cross-sectional survey. Prev Med Rep. 2019 MAR 01; 13:256-261. DOI: 10.1016/j.pmedr.2019.01.002. 2019 Jan 11. PubMed PMID: 30723659; PMCID: PMC6351349.	
Keegan THM, **Parsons HM** , Chen Y, Maguire FB, Morris CR, Parikh-Patel A, Kizer KW, Wun T. Impact of Health Insurance on Stage at Cancer Diagnosis Among Adolescents and Young Adults. J. Natl. Cancer Inst.. 2019 NOV 01; 111(11):1152-1160. DOI: 10.1093/jnci/djz039. PubMed PMID: 30937440; PMCID: PMC6855930.	
**Rosser BRS** , Kohli N, Polter EJ, Lesher L, Capistrant BD, **Konety BR (CM)**, Mitteldorf D, West W, Dewitt J, Kilian G. The Sexual Functioning of Gay and Bisexual Men Following Prostate Cancer Treatment: Results from the Restore Study. Arch Sex Behav. 2019 APR 23; . DOI: 10.1007/s10508-018-1360-y. 2019 Apr 23. PubMed PMID: 31016492.	
**Sadak KT** , Szalda D, Lindgren BR, Kinahan KE, Eshelman-Kent D, Schwartz LA, Henderson T, Freyer DR. Transitional care practices, services, and delivery in childhood cancer survivor programs: A survey study of U.S. survivorship providers. Pediatr Blood Cancer. 2019 AUG 01; 66(8):e27793. DOI: 10.1002/pbc.27793. 2019 May 16. PubMed PMID: 31099145.	
Williams LA, Frazier AL, **Poynter JN** . Survival differences by race/ethnicity among children and adolescents diagnosed with germ cell tumors. Int. J. Cancer. 2020 MAY 01; 146(9):2433-2441. DOI: 10.1002/ijc.32569. 2019 Jul 31. PubMed PMID: 31304572; PMCID: PMC6960364.	
Blair CK, Jacobs DR, Demark-Wahnefried W, Cohen HJ, Morey MC, Robien K, **Lazovich D** . Effects of cancer history on functional age and mortality. Cancer. 2019 DEC 01; 125(23):4303-4309. DOI: 10.1002/cncr.32449. 2019 Aug 16. PubMed PMID: 31418826; PMCID: PMC6856392.	
**Blaes AH** , **Shenoy C** . Is it time to include cancer in cardiovascular risk prediction tools? Lancet. 2019 SEP 21; 394(10203):986-988. DOI: 10.1016/S0140-6736(19)31886-0. 2019 Aug 20. PubMed PMID: 31443925.	
**Turcotte LM** , Liu Q, Yasui Y, Henderson TO, Gibson TM, Leisenring W, Arnold MA, Howell RM, Green DM, Armstrong GT, Robison LL, **Neglia JP** . Chemotherapy and Risk of Subsequent Malignant Neoplasms in the Childhood Cancer Survivor Study Cohort. J. Clin. Oncol.. 2019 DEC 01; 37(34):3310-3319. DOI: 10.1200/JCO.19.00129. 2019 Oct 17. PubMed PMID: 31622130; PMCID: PMC7001784.	
**2020**	
Longacre CF, Neprash HT, Shippee ND, **Tuttle TM** , **Virnig BA** . Evaluating Travel Distance to Radiation Facilities Among Rural and Urban Breast Cancer Patients in the Medicare Population. J Rural Health. 2020 JUN 01; 36(3):334-346. DOI: 10.1111/jrh.12413. 2019 Dec 17. PubMed PMID: 31846127.	
Curigliano G, Lenihan D, Fradley M, Ganatra S, Barac A, **Blaes A** , Herrmann J, Porter C, Lyon AR, Lancellotti P, Patel A, DeCara J, Mitchell J, Harrison E, Moslehi J, Witteles R, Calabro MG, Orecchia R, de Azambuja E, Zamorano JL, Krone R, Iakobishvili Z, Carver J, Armenian S, Ky B, et al. Management of cardiac disease in cancer patients throughout oncological treatment: ESMO consensus recommendations. Ann. Oncol.. 2020 FEB 01; 31(2):171-190. DOI: 10.1016/j.annonc.2019.10.023. PubMed PMID: 31959335.	
Suh E, Stratton KL, Leisenring WM, Nathan PC, Ford JS, Freyer DR, McNeer JL, Stock W, Stovall M, Krull KR, Sklar CA, **Neglia JP** , Armstrong GT, Oeffinger KC, Robison LL, Henderson TO. Late mortality and chronic health conditions in long-term survivors of early-adolescent and young adult cancers: a retrospective cohort analysis from the Childhood Cancer Survivor Study. Lancet Oncol.. 2020 MAR 01; 21(3):421-435. DOI: 10.1016/S1470-2045(19)30800-9. 2020 Feb 14. PubMed PMID: 32066543; PMCID: PMC7392388.	
Reiter PL, Gower AL, Kiss DE, Malone MA, Katz ML, Bauermeister JA, Shoben AB, Paskett ED, **McRee AL** . A Web-Based Human Papillomavirus Vaccination Intervention for Young Gay, Bisexual, and Other Men Who Have Sex With Men: Protocol for a Randomized Controlled Trial. JMIR Res Protoc. 2020 FEB 24; 9(2):e16294. DOI: 10.2196/16294. 2020 Feb 24. PubMed PMID: 32130192; PMCID: PMC7063529.	
**Thyagarajan B** , **Nelson HH** , **Poynter JN** , **Prizment AE** , Roesler MA, Cassidy E, Putnam S, Amos L, Hickle A, Reilly C, **Spector LG** , **Lazovich D** . Field Application of Digital Technologies for Health Assessment in the 10,000 Families Study. Cancer Epidemiol. Biomarkers Prev.. 2020 APR 01; 29(4):744-751. DOI: 10.1158/1055-9965.EPI-19-0858. 2020 Mar 4. PubMed PMID: 32132151.	
Yan AP, Chen Y, Henderson TO, Oeffinger KC, Hudson MM, Gibson TM, **Neglia JP** , Leisenring WM, Ness KK, Ford JS, Robison LL, Armstrong GT, Yasui Y, Nathan PC. Adherence to Surveillance for Second Malignant Neoplasms and Cardiac Dysfunction in Childhood Cancer Survivors: A Childhood Cancer Survivor Study. J. Clin. Oncol.. 2020 MAY 20; 38(15):1711-1722. DOI: 10.1200/JCO.19.01825. 2020 Mar 6. PubMed PMID: 32142393; PMCID: PMC7357338.	
**Parsons HM** , Jewett PI, **Sadak K** , **Turcotte LM** , **Vogel RI** , **Blaes AH** . e-Cigarette Use Among Young Adult Cancer Survivors Relative to the US Population. JAMA Oncol. 2020 JUN 01; 6(6):923-926. DOI: 10.1001/jamaoncol.2020.0384. PubMed PMID: 32271357; PMCID: PMC7146532.	
**Parsons HM** , Maguire FB, Morris CR, Parikh-Patel A, Brunson AM, Wun T, Keegan THM. Impact of insurance type and timing of Medicaid enrollment on survival among adolescents and young adults with cancer. Pediatr Blood Cancer. 2020 JUN 26; :e28498. DOI: 10.1002/pbc.28498. 2020 Jun 26. PubMed PMID: 32589358.	
**2021**	
**Carroll DM** , Hernandez C, Braaten G, Meier E, **Jacobson P** , Begnaud A, McGonagle E, Frizzell LB, **K Hatsukami D** . Recommendations to researchers for aiding in increasing American Indian representation in genetic research and personalized medicine. Per Med. 2021 JAN 01; 18(1):67-74. DOI: 10.2217/pme-2020-0130. 2020 Dec 17. PubMed PMID: 33332195; PMCID: PMC8242981.	
Mills LJ, **Spector LG** , **Largaespada DA** , **Williams LA** . Sex differences in expression of immune elements emerge in children, young adults and mice with osteosarcoma. Biol Sex Differ. 2021 JAN 06; 12(1):5. DOI: 10.1186/s13293-020-00347-y. 2021 Jan 6. PubMed PMID: 33407928; PMCID: PMC7789366.	
Wang S, **Prizment A** , **Thyagarajan B** , **Blaes A** . Cancer Treatment-Induced Accelerated Aging in Cancer Survivors: Biology and Assessment. Cancers (Basel). 2021 JAN 23; 13(3). DOI: 10.3390/cancers13030427. 2021 Jan 23. PubMed PMID: 33498754; PMCID: PMC7865902.	
**Vogel RI** , Yoerg B, Jewett PI, Rubin N, Olson M, Stenzel AE, Ahmed RL, **Lazovich D** . Cross-sectional study of sex differences in psychosocial quality of life of long-term melanoma survivors. Support Care Cancer. 2021 OCT 01; 29(10):5663-5671. DOI: 10.1007/s00520-021-06046-7. 2021 Feb 12. PubMed PMID: 33580285.	
**Teoh D** , Hill EK, Goldsberry W, Levine L, Novetsky A, Downs L. Overcoming the barriers to HPV vaccination in high-risk populations in the U.S.: A Society of Gynecologic Oncology (SGO) Review. Gynecol. Oncol.. 2021 APR 01; 161(1):228-235. DOI: 10.1016/j.ygyno.2021.01.023. 2021 Mar 9. PubMed PMID: 33707040.	
Moskowitz CS, Ronckers CM, Chou JF, Smith SA, Friedman DN, Barnea D, Kok JL, de Vries S, Wolden SL, Henderson TO, van der Pal HJH, Kremer LCM, **Neglia JP** , **Turcotte LM** , Howell RM, Arnold MA, Schaapveld M, Aleman B, Janus C, Versluys B, Leisenring W, Sklar CA, Begg CB, Pike MC, Armstrong GT, et al. Development and Validation of a Breast Cancer Risk Prediction Model for Childhood Cancer Survivors Treated With Chest Radiation: A Report From the Childhood Cancer Survivor Study and the Dutch Hodgkin Late Effects and LATER Cohorts. J. Clin. Oncol.. 2021 SEP 20; 39(27):3012-3021. DOI: 10.1200/JCO.20.02244. 2021 May 28. PubMed PMID: 34048292; PMCID: PMC9384912.	
**Marcotte EL** , Domingues AM, Sample JM, Richardson MR, **Spector LG** . Racial and ethnic disparities in pediatric cancer incidence among children and young adults in the United States by single year of age. Cancer. 2021 OCT 01; 127(19):3651-3663. DOI: 10.1002/cncr.33678. 2021 Jun 21. PubMed PMID: 34151418.	
Yared N, Malone M, Welo E, Mohammed I, Groene E, Flory M, Basta NE, Horvath KJ, **Kulasingam S** . Challenges related to human papillomavirus (HPV) vaccine uptake in Minnesota: clinician and stakeholder perspectives. Cancer Causes Control. 2021 OCT 01; 32(10):1107-1116. DOI: 10.1007/s10552-021-01459-5. 2021 Jul 10. PubMed PMID: 34247291; PMCID: PMC8416869.	
Groene EA, Horvath KJ, Yared N, Mohammed I, Muscoplat M, Kuramoto S, Richter T, **Kulasingam S** . Missed Opportunities for Human Papillomavirus Vaccination by Parental Nativity, Minnesota, 2015-2018. Public Health Rep. 2021 JUL 12; :333549211027244. DOI: 10.1177/00333549211027244. 2021 Jul 12. PubMed PMID: 34252324.	
**Rosser BRS** , Polter EJ, Chandiramani N, Cahill S, Wheldon CW, Konety BR, **Ryan CJ (CM)**, Haggart R, Kapoor A. Acceptability and Feasibility of Collecting Sexual Orientation and Expanded Gender Identity Data in Urology and Oncology Clinics. LGBT Health. 2021 AUG 01; 8(6):420-426. DOI: 10.1089/lgbt.2020.0256. 2021 Aug 4. PubMed PMID: 34348045; PMCID: PMC8591059.	
**Rosser BRS** , Rider GN, Kapoor A, Talley KMC, Haggart R, Kohli N, Konety BR, Mitteldorf D, Polter EJ, **Ross MW** , West W, Wheldon C, Wright M. Every urologist and oncologist should know about treating sexual and gender minority prostate cancer patients: translating research findings into clinical practice. Transl Androl Urol. 2021 JUL 01; 10(7):3208-3225. DOI: 10.21037/tau-20-1052. PubMed PMID: 34430423; PMCID: PMC8350223.	
West W, Torres MB, Mitteldorf D, Capistrant BD, Konety BR, Polter E, **Rosser BRS** . The Challenge of Coming Out to Providers by Gay and Bisexual Men With Prostate Cancer: Qualitative Results from the Restore Study. Int J Sex Health. 2021 JAN 01; 33(3):426-438. DOI: 10.1080/19317611.2021.1924335. 2021 Aug 13. PubMed PMID: 36035335; PMCID: PMC9417115.	
**2022**	
Chhikara S, Hooks M, Athwal PSS, Hughes A, Ismail MF, Joppa S, Velangi PS, Nijjar PS, **Blaes AH** , **Shenoy C** . Long-term prognostic value of right ventricular dysfunction on cardiovascular magnetic resonance imaging in anthracycline-treated cancer survivors. Eur Heart J Cardiovasc Imaging. 2022 AUG 22; 23(9):1222-1230. DOI: 10.1093/ehjci/jeab137. PubMed PMID: 34297807.	
**Gupta A** , Nshuti L, Grewal US, Sedhom R, Check DK, **Parsons HM** , **Blaes AH** , Virnig BA, Lustberg MB, Subbiah IM, Nipp RD, Dy SM, Dusetzina SB. Financial Burden of Drugs Prescribed for Cancer-Associated Symptoms. JCO Oncol Pract. 2022 FEB 01; 18(2):140-147. DOI: 10.1200/OP.21.00466. 2021 Sep 24. PubMed PMID: 34558297; PMCID: PMC9213200.	
Vivek S, **Nelson HH** , **Prizment AE** , Faul J, Crimmins EM, **Thyagarajan B** . Cross sectional association between cytomegalovirus seropositivity, inflammation and cognitive impairment in elderly cancer survivors. Cancer Causes Control. 2022 JAN 01; 33(1):81-90. DOI: 10.1007/s10552-021-01504-3. 2021 Oct 12. PubMed PMID: 34637066; PMCID: PMC8840815.	
Abdelgawad IY, Agostinucci K, **Zordoky BN** . Cardiovascular ramifications of therapy-induced endothelial cell senescence in cancer survivors. Biochim Biophys Acta Mol Basis Dis. 2022 APR 01; 1868 (4):166352. DOI: 10.1016/j.bbadis.2022.166352. 2022 Jan 15. PubMed PMID: 35041996; PMCID: PMC8844223.	
Moore KJ, **Moertel CL (IMM)**, **Williams LA** . Minority children experience a higher risk of death from many central nervous system tumor types even after accounting for treatment received: A National Cancer Database analysis. Cancer. 2022 APR 15; 128(8):1605-1615. DOI: 10.1002/cncr.34121. 2022 Feb 8. PubMed PMID: 35132615.	
**Rosser BRS** , Polter EJ, Talley KMC, Wheldon CW, Haggart R, Wright M, West W, Mitteldorf D, **Ross MW** , Konety BR, Kohli N. Health Disparities of Sexual Minority Patients Following Prostate Cancer Treatment: Results From the Restore-2 Study. Front Oncol. 2022 JAN 01; 12:812117. DOI: 10.3389/fonc.2022.812117. 2022 Feb 4. PubMed PMID: 35186749; PMCID: PMC8854183.	
Stenzel AE, Miller J, **Holtan SG** , Brown K, Ahmed RL, Lazovich D, **Vogel RI** . Cross-sectional study of physical activity among long-term melanoma survivors and population controls. Arch. Dermatol. Res.. 2022 FEB 24; . DOI: 10.1007/s00403-022-02334-2. 2022 Feb 24. PubMed PMID: 35201419; PMCID: PMC9399312.	
**Gupta A** , Eisenhauer EA, Booth CM. The Time Toxicity of Cancer Treatment. J. Clin. Oncol.. 2022 MAY 20; 40(15):1611-1615. DOI: 10.1200/JCO.21.02810. 2022 Mar 2. PubMed PMID: 35235366.	
**Nelson HH** , Contestabile E, Hunter-Schlichting D, Koestler D, Pawlita M, Waterboer T, Christensen BC, Petersen CL, **Miller JS (IMM, TCT)**, Kelsey KT. Human cytomegalovirus alters immune cell profile with potential implications for patient survival in head and neck cancer. Carcinogenesis. 2022 JUN 04; 43(5):430-436. DOI: 10.1093/carcin/bgac021. PubMed PMID: 35259245; PMCID: PMC9167029.	
Bates AJ, **Rosser BRS** , Polter EJ, Wheldon CW, Talley KMC, Haggart R, Wright M, Mitteldorf D, West W, **Ross MW** , Konety BR, Kohli N. Racial/Ethnic Differences in Health-Related Quality of Life Among Gay and Bisexual Prostate Cancer Survivors. Front Oncol. 2022 JAN 01; 12:833197. DOI: 10.3389/fonc.2022.833197. 2022 Apr 13. PubMed PMID: 35494011; PMCID: PMC9043609.	
Etteldorf A, Rotolo S, Sedhom R, **Vogel RI** , **Blaes A** , Dusetzina SB, **Virnig B** , **Gupta A** . Finding the Lowest-Cost Pharmacy for Cancer Supportive Care Medications: Not So Easy. JCO Oncol Pract. 2022 AUG 01; 18(8):e1342-e1349. DOI: 10.1200/OP.22.00051. 2022 May 27. PubMed PMID: 35623024; PMCID: PMC9377721.	
Bhatia R, **Holtan S** , **Jurdi NE** , **Prizment A** , **Blaes A** . Do Cancer and Cancer Treatments Accelerate Aging? Curr Oncol Rep. 2022 JUL 07; . DOI: 10.1007/s11912-022-01311-2. 2022 Jul 7. PubMed PMID: 35796942.	
Jewett PI, Henning-Smith C, Lazovich D, Ahmed RL, **Vogel RI** . Incidental sun exposures as a source of sunburn among rural compared to urban residents in the United States. J Rural Health. 2022 SEP 19; . DOI: 10.1111/jrh.12712. 2022 Sep 19. PubMed PMID: 36123966.	

In 2021, UMN and MCC hosted the first annual Survivorship Research Forum, dedicated to MD/PhD education and research, in collaboration with American Cancer Society, American Society of Clinical Oncology, and the National Cancer Institute (ccsrf.umn.edu). There were 236 attendees from 32 states and 5 countries. A variety of physicians, nurses, advanced practice providers, epidemiologists, and researchers were present. Three-quarters of attendees formed new collaborations, three-quarters developed new research ideas, and 95% stated the conference was “critical to cancer survivorship work” [8]. This initiative will continue to be offered biennially for the next decade to facilitate the research agenda, mentorship, and collaborations nationally among researchers, clinicians, and community partners. Additionally, several of our researchers participate in cancer survivorship leadership on a national and international level (American Society of Clinical Oncology’s Cancer Survivorship Committee, COG Late Effects team, BMT working groups). These roles allow for faculty to build research collaborations and to share research findings both nationally and locally with members of the research community and catchment area.

## Successes, challenges, and future direction

The UMN Cancer Survivorship Program has been marked by successes as well as challenges in clinical service, education, and research which are summarized in Table 3. We believe some of the key drivers to the success of our academic program include leadership buy-in and oncology integration/visibility, motivated clinical champions (who not only want to reach standards but to embrace standards), electronic health system support to increase the utility of SCPs, the leveraging of philanthropy to offset educational programming cost, collaborating with community advisory boards to engage cancer survivors and their caregivers, and the development of strong research infrastructure. On the other hand, challenges of engagement with referring providers, inconsistent guidelines for follow-up care, and finances to support a research program continue to challenge the team. Examining the successes and the gaps provides us with valuable insights, ideas, and motivations for opportunities in our program. One of our primary goals is to enhance the diversity of our patient populations; with the launch of our Community Outreach and Engagement team within MCC, we have grown community collaborations and are committed to increasing involvement for patients of all lived experience and their caregivers, such as those who live in rural areas, gender, racial, and ethnic minorities, and patients living with cancer. In addition, we aim to expand on areas of expertise within our program, such as focusing on cardio-oncology, aging, and sexual health. Finally, we aim to unify our research programs leveraging research programs into a center focused on cancer survivorship across the lifespan integrating patient reported outcomes, home health metrics, and specific aspects that face cancer survivors such as accelerated aging. By harnessing our challenges and embracing new ideas, we can further enhance our cancer survivorship program and make a positive impact on the lives of our patients.

**Table 3:** Successes, challenges, and future directions

	Successes	Challenges	Future direction
Clinical	• Unique model that prioritizes individualized cancer survivorship care across the lifespan • Separate survivorship groups working together in a team approach (childhood, adult, BMT) • Leadership buy-in • Dedicated MD/APP champions and program manager • Electronic health system support • COVID-19 pandemic served as a catalyst for the rapid adoption of virtual care, which has reduced geographical barriers to care	• Engagement of primary oncologists and primary care in utilizing cancer survivorship and smooth transitions • Lack of consistent guidelines in adult cancer survivors • Translating the action items of the SCP into provider orders • During the early COVID-19 pandemic, services were temporarily paused as they were considered lower priority	• Introduce cancer survivorship early in the cancer continuum • Enhance the diversity of our patient populations, such as those who live in rural areas, gender, racial, and ethnic minorities, and patients living with cancer by working community outreach and engagement • Expand on areas of expertise within our program, such as focusing on cardio-oncology, aging, and sexual health • Implementing a learning health-care system to enhance efficiency and accessibility for our patients
Education	• Leveraging of community and philanthropic partnerships has increased patient resources, shared knowledge, enhanced community engagement and increased reach and impact • Unique educational programs for researchers, medical trainees, clinical workforce (RNs, APPs, MDs)	• Organization of patient education materials on several web pages across our research programs and clinical partners	• Creating centralized e-learning modules for our patients • Centralizing educational programs for clinical staff
Research	• Bidirectional research infrastructure is well-established addressing research questions raised by patients, allowing patient participation in research activities on multiple levels	• Challenges compiling data across programs due to the differing data points collected by each program • Financial support to leverage databases and research teams	• Utilizing consistent tools to measure outcomes across the program to track progress • Leveraging research programs to establish a Research Center for Cancer Survivorship Across the Lifespan
